# Optimization of Buffalo (*Bubalus bubalis*) Embryonic Stem Cell Culture System

**DOI:** 10.22074/cellj.2016.3728

**Published:** 2015-07-11

**Authors:** Mohammad Zandi, Musharifa Muzaffar, Syed Mohmad Shah, Manoj Kumar Singh, Prabhat Palta, Suresh Kumar Singla, Radheysham Manik, Manmohan Singh Chauhan

**Affiliations:** 1 Department of Animal and Poultry Science and Fisheries, Agricultural Institute, Iranian Research Organisation for Science and Technology (IROST), Tehran, Iran; 2 Embryo Biotechnology Laboratory, Animal Biotechnology Centre, National Dairy Research Institute (NDRI), Karnal, India

**Keywords:** Buffalo, Embryonic Stem Cells, Y-27632, FGF-2, LIF

## Abstract

**Objective:**

In order to retain an undifferentiated pluripotent state, embryonic stem (ES) cells have to be cultured on feeder cell layers. However, use of feeder layers limits stem cell research, since experimental data may result from a combined ES cell and feeder cell response to various stimuli.

**Materials and Methods:**

In this experimental study, a buffalo ES cell line was established from *in vitro* derived blastocysts and characterized by the Alkaline phosphatase (AP) and immunoflourescence staining of various pluripotency markers. We examined the effect of various factors like fibroblast growth factor 2 (FGF-2), leukemia inhibitory factor (LIF) and Y-27632 to support the growth and maintenance of bubaline ES cells on gelatin coated dishes, in order to establish feeder free culture systems. We also analyzed the effect of feeder-conditioned media on stem cell growth in gelatin based cultures both in the presence as well as in the absence of the growth factors.

**Results:**

The results showed that Y-27632, in the presence of FGF-2 and LIF, resulted in higher colony growth and increased expression of Nanog gene. Feeder-Conditioned Medium resulted in a significant increase in growth of buffalo ES cells on gelatin coated plates, however, feeder layer based cultures produced better results than gelatin based cultures. Feeder layers from buffalo fetal fibroblast cells can support buffalo ES cells for more than two years.

**Conclusion:**

We developed a feeder free culture system that can maintain buffalo ES cells in the short term, as well as feeder layer based culture that can support the long term maintenance of buffalo ES cells.

## Introduction

Buffalo embryonic stem (ES) cells, derived from the inner cell mass (ICM) of blastocysts, can be maintained in culture under conditions that retain their pluripotency (1). Research on buffalo ES cells has elucidated important signaling pathways, the wingless-type MMTV integration site family, member 3A (WNT3A), bone morphogenetic protein 4 (BMP-4), transforming growth factor beta (TGF-β)/activin/nodal pathways, that play a role in maintaining pluripotency or differentiation (2, 3). However, there is not much information available on the culture of buffalo ES cells and the extrinsic and intrinsic factors that affect their culture. In order to retain an undifferentiated pluripotent state, ES cells need to be cultured on buffalo or murine feeder cell layers (4). However, the use of feeder layers limits stem cell research, since experimental data may result from a combined ES cell and feeder cell response to various stimuli. To overcome this problem, some studies have described a system for feeder-free and conditioned media (CM) free culture of human ES cells, even though these ES cells have been exposed to feeder cells during derivation (5). In 2001, Xu et al. (6), compared the culture of various WiCell lineson Matrigel, laminin, fibronectin and collagen IV in the presence of mouse embryonic fibroblast (MEF)-CM. They found that the cells survived poorly and differentiated rapidly when cultured on gelatin, but both laminin and Matrigel were able to support undifferentiated growth of the human ES cells (7). However, the use of such matrix increases the expense of research in animal field.

Conditioned media from MEF cells can support the self-renewal of mouse ES cells, eliminating the need for a feeder layer. It was demonstrated that mouse EF cells inhibit ES cell differentiation via production of the interleukin-6 family cytokine, leukemia inhibitory factor (LIF). With the addition of recombinant LIF protein into the culture medium, mouse ES cells can be cultured without mouse EF cells (8). LIF binds the heterodimeric LIF receptor-glycoprotein 130 complex and activates Jak kinases with recruitment of Shp-2 and signal transducer and activator of transcription 3 (STAT3) (9). Unlike mouse ES cells, extrinsic factor LIF is not sufficient to maintain human ES cells. Instead, fibroblast growth factor (FGF) signaling is central to the self-renewal of human ES cells (10, 11). The FGF-2 signals are transduced through receptors with intrinsic protein tyrosine kinase activity (12). Virtually all human ES cell media described to date contain FGF-2. FGF-2 supplementation has been associated with pleiotropic-positive effects: impeding spontaneous differentiation, increasing human ES cell proliferation, enhancing attachment/survival, inhibiting earliest neural induction, and, more precisely, moderately stimulating Nanog gene expression. In contrast, the FGF/extracellular-signal-related kinase cascade plays a role in the differentiation of mouse ES cells (13).

In an effort to circumvent the problem of apoptosis in human ES cell culture, Watanabe et al. (14) showed that addition of a selective Rho-associated kinase (ROCK) inhibitor, Y-27632, to the human ES cell medium increased colony formation of dissociated human ES cells, possibly through the regulation of myosin light chain (MLC) phosphorylation and cell-cell interactions (15, 16). ROCK inhibitor, Y27632, supports human ES cell culture in a matrice-free environment (17) and significantly increases the survival of human ES cells after thawing, compared with that of the control group (18). More recently, Claassen et al. (19) determined that Y-27632 significantly improves the recovery of cryopreserved human induced pluripotent cells and their growth on subculture.

We examined the effect of a chemically-defined environment, consisting of FGF-2, LIF and Y-27632 in gelatin coated dishes, on the short term maintenance of buffalo ES cells. We also evaluated the growth of buffalo ES cells on a buffalo fetal fibroblast (FF) feeder layer and gelatin coated dishes with and without conditioned media derived from buffalo FF feeder layer.

## Materials and Methods

This experimental study was approved by the Ethics Committee of the Embryo Biotechnology Lab, National Dairy Research Institute (NDRI, Karnal, India). Unless mentioned otherwise, all culture media, growth factors, fetal bovine serum (FBS), and other chemicals were purchased from Sigma (St. Louis, MO, USA), and plastics were purchased from Falcon (Paignton, UK).

### In vitro embryo production

Buffalo ovaries were obtained from the Delhi slaughter house and transported to the laboratory in phosphate-buffered saline containing penicillin (100 IU/mL) and streptomycin (50 mg/mL) at 30-34˚C within 5 hours of slaughter. Cumulus-oocyte complexes (COCs), from follicles 2-8 mm in diameter, were aspirated using an 18 G needle attached to a 10 mL disposable syringe. A group of fifteen to twenty excellent quality COCs were transferred to a 100 mL droplet of the *in vitro* maturation (IVM) medium (TCM 199+10% FBS+5 μg/ mL porcine follicle stimulating hormone (pFSH)+1 μg/mL estradiol-17β+0.81 mM sodium pyruvate+5-10% buffalo follicular fluid+50 μg/mL gentamicin sulfate) under mineral oil in a petridish and cultured at 38.5˚C in a humidified atmosphere of 5% CO_2_ for 24 hours. The *in vitro* matured oocytes were washed twice with Bracket and Oliphant’s (BO) medium and transferred to 50 μL droplets (15-20 oocytes/droplet) of the medium. The spermatozoa were prepared for fertilization as per the protocol established by Chauhan et al. (20). Oocytes were then inseminated by addition of spermatozoa at the final concentration of 1.0-2.0×10^6^ motile sperm/ mL. Sperm and oocytes were incubated under paraffin oil at 39˚C under a humidified atmosphere of 5% CO_2_ for 18 hours. At the end of the insemination period, groups of ten oocytes were stripped free from cumulus cells and transferred into modified Charles Rosenkrans medium with amino acids (mCR2aa) containing 0.6% bovine serum albumin (BSA). The cells were cultured in this medium for the first 2 days, then transferred to IVC medium (mCR2aa+0.6% BSA+10% FBS). The culture medium was changed every 2 days upto 8 days, till the blastocysts were obtained.

### Establishment of buffalo embryonic stem cells

Buffalo ES cells were derived from *in vitro* fertilized embryos as described by Muzaffar et al. (1). Briefly the inner cell mass from the embryos was dissected out and seeded overmitomycin-C treated buffalo fetal fibroblast cells in ES medium consisting of Knockout Dulbecco’s Modified Eagle Medium (KO-DMEM, GIBCO/BRL) supplemented with 15% knockout serum replacement medium (GIBCO/BRL), 2 mM L-glutamine, 0.1 mM β-mercaptoethanol, 1% nonessential amino acids (all from GIBCO/BRL), 1000 U/ mL LIF, and 5 ng/ mL FGF-2 (R & D Systems). The media was changed every alternate day and passaging was performed after 7 days.

### Conditioning medium

Mitotically inactivated buffalo FF cells (treated with 10 μg/ml mitomycin-C) were cultured in T-25 flasks (Iwaki) with addition of ES medium for 7 days. Buffalo fibroblast-conditioned medium was collected every day, centrifuged at 200 g for 3 minutes, filtered with a 0.2 μm syringe filter (Millipore, Watford, England) and frozen at -80˚C. After thawing, the medium was equilibrated for 2 hours at 5% CO_2_ and 37˚C and then used for the feederfree culture of human ES cells.

### Characterization of the stem cells

Alkaline phosphatase (AP) and immunofluorescence were used for characterization of buffalo ES cells. The cell surface antigens used for characterization were the glycolipids stage-specific embryonic antigen-1 (SSEA-1) and SSEA-4, the keratan sulfate antigens tumor rejection antigen-1-60 (TRA-1-60) and TRA-1-81 (Chemicon, Millipore, Cat NoSCR002) and the pluripotency markers NANOG (Santa Cruz, Cat No. SC134218), OCT3/4 (Chemicon, Millipore, Cat NoSCR002) and SOX2 (Chemicon, Millipore, Cat No. SC1002).

### RNA isolation, reverse transcriptionand quantitative real-time polymerase chain reaction (qPCR)

Total RNA was isolated with Trizol reagent (Invitrogen) and subsequently treated with DNAse (Ambion, Woodlands, TX) to avoid DNA contamination. Reverse transcription was done with Moloney Murine Leukemia Virus Reverse Transcriptase (MMLV) enzyme (USB) and oligo dT priming. qPCR was carried out with SYBR Green mix (ABI). Calculations were based on the ΔΔCt method employing *Gapdh* for normalization. Primer sequences are listed in table 1.

### Experimental design

In experiment 1, the effect of FGF-2 (5 ng/ mL) and LIF (1000 U/ml), on stem cell growth, in gelatin coated dishes was analyzed. This experiment consisted of three treatments and control groups: i. ES cell medium (KO-DMEM supplemented with 15% KSR), 2 mM nonessential amino acids, 2 mM L-glutamine, 50 μg/ml gentamicin, 0.1 mM β-mercaptoethanol) as control, ii. ES cell medium+FGF-2 (5 ng/mL), iii. ES cell medium+LIF (1000 U/ml) and iv. ES cell medium+FGF-2 (5 ng/mL)+LIF (1000 U/ml).

In experiment 1, the effect of FGF-2 (5 ng/ mL) and LIF (1000 U/ml), on stem cell growth, in gelatin coated dishes was analyzed. This experiment consisted of three treatments and control groups: i. ES cell medium (KO-DMEM supplemented with 15% KSR), 2 mM nonessential amino acids, 2 mM L-glutamine, 50 μg/ml gentamicin, 0.1 mM β-mercaptoethanol) as control, ii. ES cell medium+FGF-2 (5 ng/mL), iii. ES cell medium+LIF (1000 U/ml) and iv. ES cell medium+FGF-2 (5 ng/mL)+LIF (1000 U/ml).

In experiment 3, the effects of buffalo FF feeder layer, gelatin coated dishes, and gelatin coated dishes with feeder-CM on the growth of buffalo ES cells were compared. In this experiment ES cell medium contained FGF-2 (5 ng/mL)+LIF (1000 U/ ml)+Y-27632(10 μM). This experiment was performed with five treatments: i. gelatin coated dishes, ii. normal concentration of fibroblast cells in feeder layer (3×10^4^ cells/cm^2^), iii. high concentration of fibroblast cells in feeder layer (5×10^4^ cells/cm^2^), iv. 50% feeder-CM in gelatin coated dishes and v. 100% feeder-CM in gelatin coated dishes.

**Table 1:** Primers for real-time polymerase chain reaction


Gene	Sequence	Annealing temperature	Base pairs	Acc. No

Sox2	F: 5΄CGTGGTTACCTCTTCTTCC3΄	60	139	GQ85388
	R: 5΄CTGGTAGTGCTGGGACAT3΄			
Oct3/4	F: 5΄TTGCAGCTCAGTTTCAAG3΄	54	75	EU926737
	R: 5΄GTTGTTGTCAGCTTCCTC3΄			
Nanog	F: 5΄CCGAAGCATCCAACTCTAGG3΄	60	100	NM001025344.1
	R: 5΄GAGACAGTGTCCGTGTCGAG3΄			
C-myc	F: 5΄CTCCTCACAGCCCGTTAGTC3΄	53	156	GU296437.1
	R: 5΄ATTTGCGGTTGTTGCCTATC3΄			
Gapdh	F: 5΄TCAAGAAGGTGGTGAAGCAG3΄	57	121	GU324291.1
	R: 5΄CCCAGCATCGAAGGTAGAAG3΄			


### Statistical analysis

Comparisons between multiple numeric data sets were performed using one-way ANOVA followed by the Duncan multiple range test. Results were expressed as mean ± standard error of the mean (SEM), and statistical significance was accepted for P<0.05. Data were analyzed with a statistical software program SPSS (SPSS 11.5, 2004, IBM, USA).

## Results

### Establishment of embryonic stem cell like cells from blastocysts produced through in vitro fertilization

**Fig.1:**
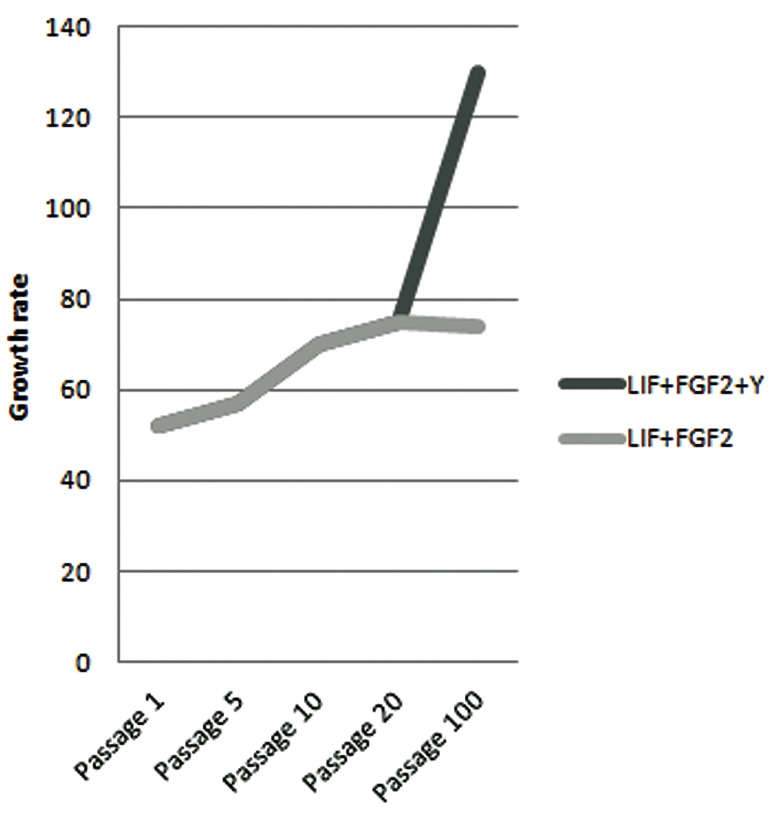
The growth rate of buffalo ES cell colonies in the presence of LIF and FGF-2 either alone or in the presence of Y-27632 (Y=Y- 27632). ES; Embryonic stem, LIF; Leukemia inhibitory factor and FGF-2; Fibroblast growth factor.

ES cell like cells were established from preimplantation stage embryos (blastocysts) by dissecting out ICM which was cultured over mitomycin C treated buffalo fetal fibroblast cells in the presence of growth factors. ICM when seeded on feeder layer cells resulted in outgrowths or primary colonies, for which attachment of ICMs to feeders is the most important criterion. ICMs were found to be attached to the feeder layer by day 3 and the time taken for primary colony formation was 8 to 12 days. From a total of 86 ICM, the primary colony formation rate was 52% (Table 2). The growth rates of ES cells by the number of colonies after each passage was around 57% till 5th passage and reached 70 and 75% by the10^th^ and 20^th^ passages, respectively, in the presence of LIF and FGF-2. Addition of Y-27632 in combination with LIF and FGF-2 to ES cell cultures caused a 56% increase in the growth rate of ES cell colonies at passage 100 compared with LIF and FGF-2 alone (Fig.1).

**Table 2:** ICM derived primary colony formation rates


Maximumpassagenumber	ICMs seeded (n)	Time taken forprimarycolony formation(days)	Primary colonyformation rate(Mean ± SEM)

100	86	8-12	52.44 ± 2.55


ICM; Inner cell mass, n; Number and SEM; Standard error of mean.

### Characterization of buffalo embryonic stem cells

**Fig.2:**
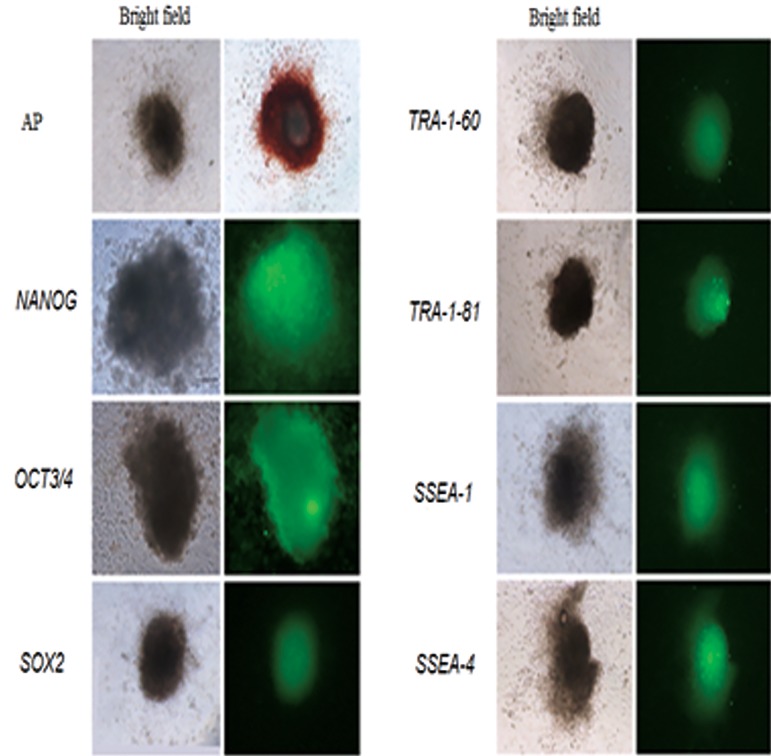
Alkaline phosphatase and immunofluorescence staining for characterization of buffalo ES cells at passage 20. AP; Alkaline phosphatase, ES; Embryonic stem, SSEA; Stage-specific embryonic antigen and TRA; Tumor rejection antigen.

At regular intervals the colonies were characterized using different ES cell markers. The ES colonies were positive for SSEA-1, SSEA-4, TRA-1- 60 and TRA-1-81, as well as for NANOG, OCT3/4 and SOX2 at passage 20 (Fig.2).

**Fig.3:**
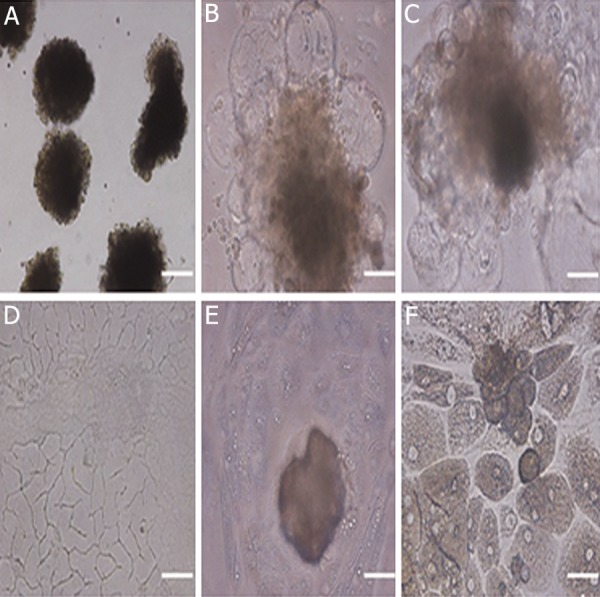
A. Compact embryoid bodies, B, C. Cystic embryoid bodies produced from buffalo ES cells, D, E and F. Different cell types produced by spontaneous differentiation (lipid-like cells, epithelial- like cells and hepatocyte-like cells, respectively, scale bar=200 μm). ES; Embryonic stem.

To test the differentiation potential of the ES cells, colonies were dissected mechanically into small clumps of 400-1000 cells and cultured in 20- to 30- μL hanging drops of DMEM and 15% knockout serum replacement (KSR) in bacterial dishes in the absence of the growth factors and feeder cells for embryoid bodies (EB) formation. This led to differentiation of the ES cells to form three-dimensional round aggregates. Compact EBs were formed within 2-4 days (Fig.3A) and developed to cystic EBs, when these cultures were maintained for 1-2 weeks (Fig.3B, C). It was observed that spontaneous differentiation of buffalo ES cells for 20 days resulted in the formation of different types of cells, such as lipid-like cells, epithelial-like cells and hepatocyte-like cells, etc (Fig.3D-F).

### Experiment 1. The effect of fibroblast growth factor 2, leukemia inhibitory factor in gelatin coat based culture on buffalo embryonic stem cells

**Fig.4:**
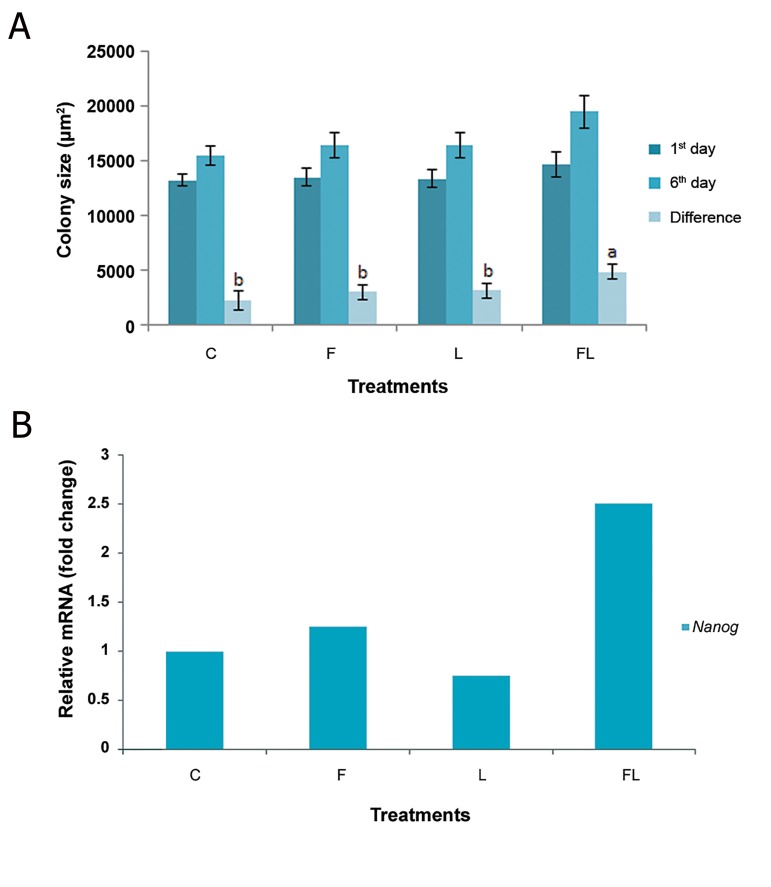
The effects of fibroblast growth factor 2 (FGF-2, 5 ng/ml) and leukemia inhibitory factor (LIF, 1000 U) on buffalo ES cells cultured on gelatin coated dishes on, A. Mean area of buffalo ES colonies and B. Expression of pluripotency genes. C; Control, F; FGF-2, L; LIF, FL; FGF-2+LIF and ES; Embryonic stem.

It was observed that the supplementation of ES cell medium with either FGF-2 (5 ng/ml) or LIF (1000 U) in gelatin coat based culture, did not increase the growth of buffalo ES cells whereas, the combination of FGF-2 and LIF (FGF-2+LIF) significantly (P<0.05) increased the growth of buffalo ES cell colonies (Fig.4A). Real-time PCR data showed that the expression of Nanog gene was higher for buffalo ES cell colonies in medium supplemented with FGF-2+LIF (Fig.4B).

### Experiment 2. The effect of rock inhibitor (Y-27632) in gelatin coat based culture on buffalo embryonic stem cells

**Fig.5:**
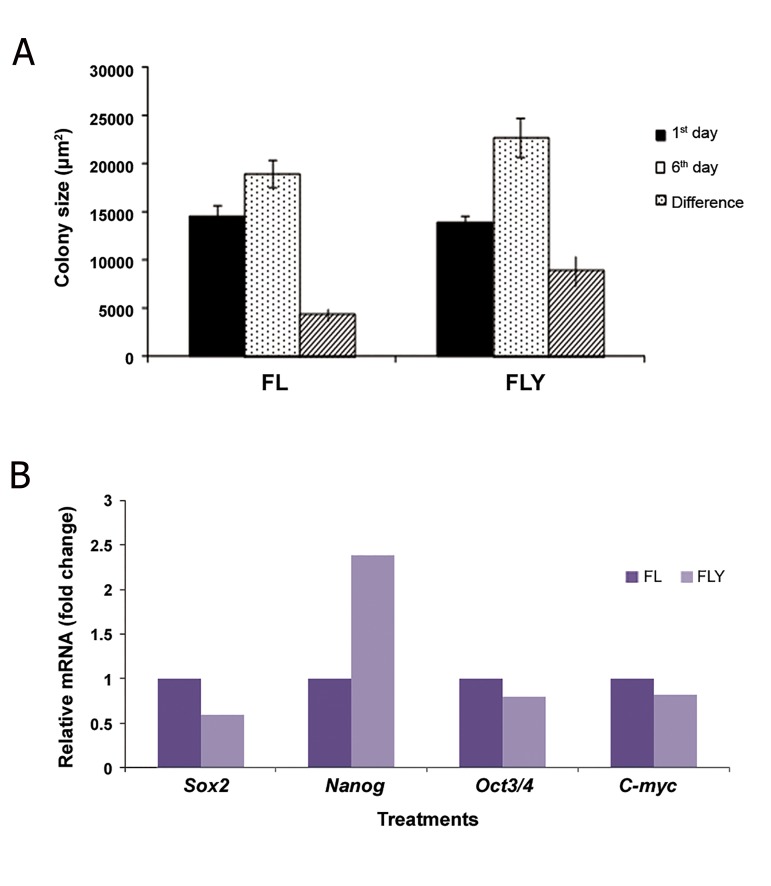
The effects of Y-27632 (10 μM) on buffalo ES cells cultured on gelatin coated dishes on A. Mean area of buffalo ES cellcolonies and B. Expression of pluripotency genes. FGF-2; Fibroblast growth factor 2, LIF; Leukemia inhibitory factor, FL; FGF-2+LIF, FLY; FGF-2+LIF+Y-27632 and ES; Embryonic stem.

The effect of Y-27632 supplementation of ES cell medium supplemented with FGF-2 and LIF on gelatin coated dishes on the mean area of ES cells and expression of pluripotency genes are summarized in figure 5. Supplementing the ES cell medium with 10 μM Y-27632 significantly (P<0.05) affects the mean area of buffalo ES cells (Fig.5A). Real-time PCR analysis showed that the Nanog gene was up-regulated in the presence of Y-27632 as compared to the control (Fig.5B).

### Experiment 3. Comparison between feeder layer based culture and gelatin coat based culture, either alone or in the presence of feeder-conditioned media

**Fig.6:**
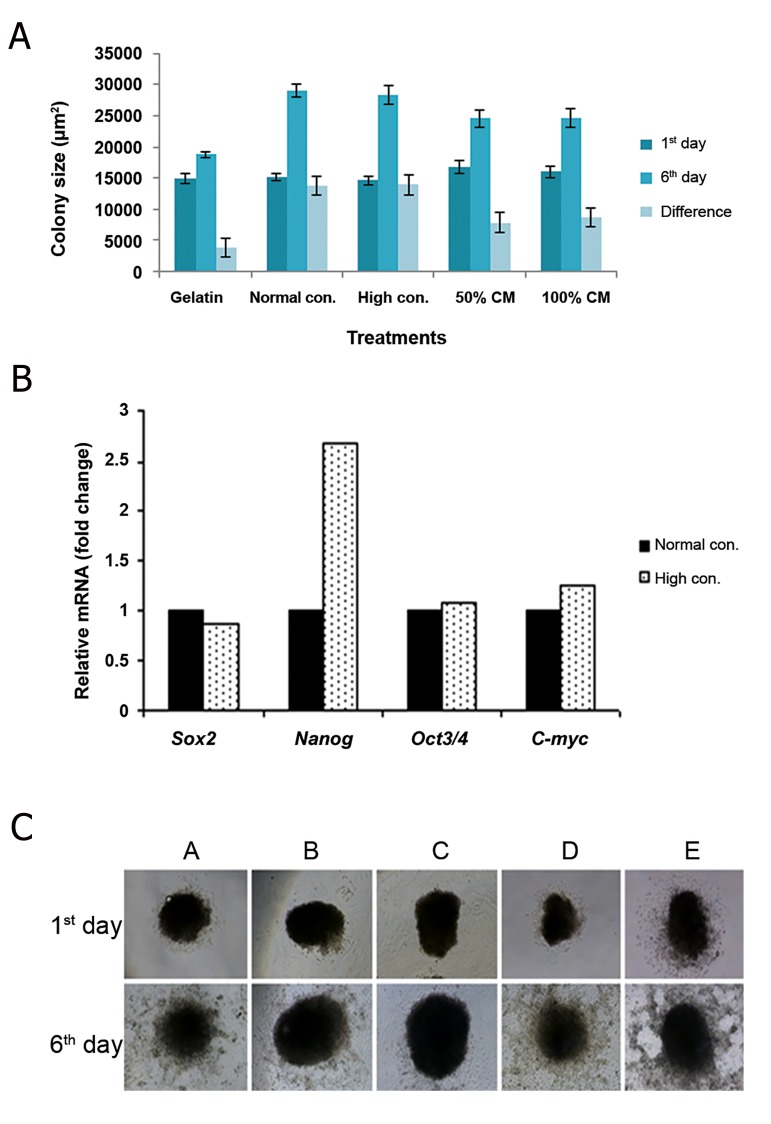
The effects of bFF feeder layer and gelatin coated dishes either alone or feeder-CM, on buffalo ES cell colonies. A. Mean area of buffalo ES cellcolonies, B. Expression of pluripotency genes for normal and high concentration of fibroblast cells (3x10^4^ and 5x10^4^ cells per cm^2^, respectively) in feeder layer and C. Phase-contrast image after 24 hours and 6 days of culture, A. Gelatin coated dishes, B. Normal concentration of bFF cells in feeder layer, C. High concentration of bFF cells in feeder layer, D. 50% feeder-CM in gelatin coated dishes and E. 100% feeder-CM in gelatin coated dishes. bFF; Buffalo Fetal fibroblast, CM; Conditioned media and ES; Embryonic stem.

Feeder layers from normal and high concentration of fibroblast cells (3×10^4^ and 5×10^4^ cells per cm^2^, respectively) resulted in significantly (P<0.05) higher growth of buffalo ES cells than gelatin coated dishes, either alone or in the presence of feeder-CM (from buffalo FF feeder cells). However, no statistically significant difference was evident when comparing feeder layers from a normal or high concentration of buffalo FF cells. The mean area of buffalo ES cells was significantly increased after culture in the presence of feeder- CM (P<0.05) as compared to gelatin coated dishes alone (Fig.6A), real-time PCR analysis, as shown in figure 6B, revealed that a higher concentration of fibroblast cells in the feeder layer resulted in higher expression of Nanog in buffalo ES cells, compared to a normal concentration of fibroblast cells. Feeder layer from buffalo fetal fibroblast cells can support buffalo ES cells for more than two years. Whereas, in the presence of feeder-CM, the ES cells spread more in culture, a sign of differentiation (Fig.6C).

## Discussion

The combination of FGF-2+LIF significantly (P<0.05) increased the growth of buffalo ES cell. The expression of Nanog gene was also higher for buffalo ES cell colonies in medium supplemented with FGF-2+LIF.The results are in agreement with that of Sharma et al. (21) who showed that the buffalo ES cell colony size was highest following supplementation with FGF-2 and LIF. They also showed that FGF-2 supplementation affected the quantitative expression of Nanog and Sox-2, but not Oct3/4. The synergistic effect between FGF-2 and LIF, as observed in our studies, in supporting growth and pluripotency of ES cells was specific to buffalo ES cells only and not seen in human and mouse ES cells. Xu et al. (22) observed that FGF-2 alone or in combination with other growth factors supports human ES cell growth, while LIF without FGF-2 was not sufficient to maintain the growth of undifferentiated cells. The active and direct role of FGF-2 signaling in supporting selfrenewal through Nanog is specific to human ES cells and is not seen in mouse epiblast stem cells (EpiSCs). The shear existence of a human ES cellspecific feature is, in principle, not very surprising considering that human ES cells are equivalent to neither mouse ES cells nor to EpiSCs and there are notable differences in early development between mouse and human (13). More importantly, FGF-2 has recently been shown to be sufficient to support human ES cell growth on MatrigelTM in the absence of feeders or feeder-CM (23). Wang et al. (24) suggested that FGF-2 most likely represents a signaling pathway similar to LIF for mouse ES cells. Daheron et al. (25) further observed the lack of cross-reactivity in murine and human forms of LIF for the LIF receptor.

The addition of Y-27632 to gelatin coated based culture, in the presence of FGF-2 and LIF, significantly (P<0.05) improved the growth of buffalo ES cells. Our result is in agreement with the studies conducted by Harb et al. (26) who showed that human ES cells can be grown without the need for niche-forming feeder layers or animal-derived matrices with the addition of Y-27632 in a single synthetic matrix, i.e. poly-D-lysin. It has been showed that Y-27632 augments survival of human ES cells not only by decreasing the level of apoptosis but also through complementary mechanisms, such as increasing cellular adhesion by promoting stronger cell-cell interaction. Rho accepts signals from G-protein-coupled receptors in addition to other signaling pathways that originate in the extracellular matrix (ECM), as well as intracellularly. Rho activation of ROCK leads to the phosphorylation of a number of downstream targets which are involved in diverse signaling pathways (15). Li et al. (27) showed that ROCK protein transduces signals from the cortical actin cytoskeleton and ECM to the nucleus, leading to changes in cell morphology as well as transcriptional regulation. Thus, by interrupting signals from the cellular environment by inhibiting ROCK, human ES cells are no longer aware of their current environment. Peerani et al. (28) demonstrated that Y-27632 treatment increased levels of Oct3/4 expression. However, our studies revealed that Y-27632 in feeder independent culture resulted in the up-regulation of Nanog gene.

The results showed that presence of feeder-CM in gelatin coat based culture significantly improved the growth of buffalo ES cells compared to gelatin coat based culture alone. Lim and Bodnar (29) showed that CM from mouse embryonic fibroblast feeder layers contains 136 unique protein species which included some that are known to participate in cell growth and differentiation, extracellular matrix formation, and remodeling, in addition to the unexpected but interesting finding of many nominally intracellular proteins. However, gelatin coat based cultures conditioned with buffalo FF feeder layer were not adequate to support the growth of buffalo ES cells compared with feeder layer based cultures. Thus, buffalo ES cells are dependent upon matrix for proliferation and maintenance. It is established that the feeder cells provide secretory factors, extracellular matrix, and cellular contacts for the maintenance of ES cells in the undifferentiated state without losing pluripotency (30). The results of our study showed that a higher concentration of feeder-CM (100 vs. 50%) in feeder free culture resulted in more ES cell spreadingon a gelatin coated plate, while a higher concentration of fibroblast cells in a feeder layer based culture improved the expression of Nanog gene in ES cells.

## Conclusion

*In vitro* culture conditions are not just supportive but also instructive and their being instructive is a major concern for ES cell cultures which have the ability to differentiate into all three germ layers. Both the nature and potential of differentiation is modified by the culture niche which plays a critical role in stem cell cultures due to their dependence on the feeder layer. In order to optimize feeder free culture conditions, we investigated the effect of various growth factors like FGF, LIF and Y-27632 on both feeder based as well as feeder free conditions. Gelatin coated dishes were used as an alternative to the feeder based system for ES cell culture and it was observed that they could serve the purpose, especially when feeder cells could be viewed as contaminant cells for some specialized studies. The study implies that gelatin-based culture could be used for short term culture and propagation of buffalo Es cells, while feeder based cultures are better for long term purposes. It can also be presumed that gelatin coated dishes could alsoin practice be useful for long term cultures, albeit after further culture characterization.
